# A Novel Puff Recording Electronic Nicotine Delivery System for Assessing Naturalistic Puff Topography and Nicotine Consumption During Ad Libitum Use: Ancillary Study

**DOI:** 10.2196/42544

**Published:** 2023-01-16

**Authors:** Xiang Gao, Melody Fewx, John Sprock, Huanhuan Jiang, Yong Gao, Yatao Liu

**Affiliations:** 1 Scientific Horizons Consulting Irvine, CA United States

**Keywords:** electronic nicotine delivery system, electronic cigarettes, puff topography, nicotine consumption, ad libitum use

## Abstract

**Background:**

Assessing the naturalistic puff topography and associated nicotine consumption during e-cigarette use is important as such information will not only unveil how these products are being consumed in real-world conditions, but also enable investigators and regulatory bodies to conduct quantitative, accurate, and realistic harmful exposure and nicotine abuse liability risk assessments based on actual e-cigarette use. Conventional approaches cannot accurately, conveniently, and noninvasively determine e-cigarette puff topography in a natural use environment. Thus, novel technology-enabled systems that do not primarily rely on self-report mechanisms or intrusive measurements to monitor e-cigarette product use behaviors are highly desired.

**Objective:**

This study aimed to explore and demonstrate the feasibility of a novel puff recording electronic nicotine delivery system (PR-ENDS) device for measuring naturalistic puff topography and estimating nicotine consumption during the ad libitum use of products among smokers and vapers.

**Methods:**

An ancillary data analysis based on a completed parent study was conducted. The parent study was a 1-way randomized controlled open-label puff topography and nicotine pharmacokinetic assessment carried out in 24 healthy adults (12 smokers and 12 vapers). Participants were assigned a randomized product use sequence of a PR-ENDS device within 5 site visits for both controlled and ad libitum product use sessions. Blood samples were obtained for plasma nicotine analysis, and questionnaires were administered at various time points. During the ad libitum use session, puff topography was measured using a Clinical Research Support System (CReSS) device as a benchmark, as well as the PR-ENDS device with a built-in puff recording feature.

**Results:**

There were no significant differences in representative puff topography parameters (number of puffs, total puff duration, and average puff duration) between the PR-ENDS and CReSS devices at the populational level across different device powers, e-liquid nicotine strengths, and flavors. The nicotine consumption estimated by the PR-ENDS device suggested that this device can be employed as a convenient monitoring tool for estimating nicotine use without measuring e-liquid weight loss between puffs. The linear relationship between nicotine consumption estimated by the PR-ENDS device and the pharmacokinetic parameter AUC_ad lib_ (plasma concentration-time curve for 1-hour ad libitum use) substantiated the potential of using this device as a pragmatic, noninvasive, and convenient means for estimating nicotine intake in the human body without blood collection.

**Conclusions:**

The novel PR-ENDS device was feasible for assessing naturalistic puff topography and estimating nicotine consumption and intake in the human body during ad libitum use. Several key factors can influence users’ puff topography, including device power levels, e-liquid nicotine strengths, and flavors. The study results pave the way for further research in the real-time measurement of naturalistic puff topography and puffing behaviors in the real world.

## Introduction

Electronic nicotine delivery systems (ENDSs) or electronic cigarettes (e-cigarettes) have become a global public health concern, with a significant increase in prevalence in the past decade [1-3]. The use of e-cigarettes is considered controversial in 2 aspects. First, e-cigarettes are not harm-free, and the long-term health effect is yet to be evaluated [4-7]. E-cigarettes are not for nonsmokers, especially adolescents, as nicotine is highly addictive and can harm adolescent brain development [8] and function, with a gateway effect for other addictions such as combustible cigarettes, alcohol, and marijuana [9-11]. Second, e-cigarettes have the potential to benefit current adult smokers if used as a complete substitute for cigarettes and other smoked tobacco products owing to the significantly reduced toxicant emissions compared to combustible cigarettes [12,13]. In general, aerosols produced from e-cigarettes contain fewer and much lower levels of harmful constituents and carcinogens compared to that in combustible cigarettes [14-17], which renders e-cigarettes a potential candidate for tobacco harm reduction. Yet, the health effects of e-cigarettes are rather complex, and not all puffs expose e-cigarette users to the same emission levels [18-21]. Within such a context, the assessment of e-cigarette representative puff topography (ie, number of puffs, puff duration, puff interval, etc), especially in a natural environment and noninterventional condition, becomes extremely important because (1) puff topography directly reflects the nicotine usage of e-cigarettes, such as nicotine consumption, which can provide a quantifiable scale to estimate the degree of nicotine addiction and abuse liability; (2) puff topography provides a quantifiable basis to assess exposure to harmful and potentially harmful constituents in individual health risk assessment [22-24]; and (3) direct measurement of puffing behavior assists in revealing product use patterns and potential correlations with e-cigarette attributes, such as e-liquids and devices, and such information can be of great value in directing product design and regulations for improved tobacco harm reduction.

So far, various techniques and strategies have been applied to measure the puff topography of e-cigarette use. The most commonly recognized method is the self-reporting survey [25-28], in which users are asked a series of questions regarding their e-cigarette use behaviors, including frequency and intensity of use. The limitation of this approach is rather obvious, as the data collected are self-reported and can easily be subject to recall and response bias. Another technique commonly used for e-cigarette use behavior is the video assessment method, where investigators assess video recordings of a user’s puff start and end times frame-by-frame to determine the puff duration and interpuff interval [29-32]. Video assessment partially addresses the problems in the self-reporting survey, in that the investigation from the surveillance camera can validate user behaviors and provide quantitative data for analysis. However, video assessment can be problematic when there is a lack of visual cues (ie, LED light on/off, facial expression, etc) [32]. The presence of surveillance cameras might also lead to alterations in typical behaviors. In addition, video assessment is conducted by filming the entire use session, normally followed by a time-consuming data processing step. These difficulties limit the practical application of video assessment in the real world. Lastly, several specialized puff-sensing devices have been developed in the research field to monitor e-cigarette user’s puffing behaviors, such as Clinical Research Support System (CReSS) [33-35], Smoking Puff Analyzer Mobile Device (SPA-M) [36,37], and Wireless Portable Use Monitor (wPUM) [18,38,39]. Although they are widely used in the research field, a noticeable drawback of such techniques is their restricted applicability in laboratory and clinical environments. The use of these specialized devices becomes extremely costly in a real-world condition and in a large study group setting owing to cost and compliance issues [40]. The additional mouthpiece adaptor fixed on the e-cigarette may also make the usage experience different from that of direct e-cigarette use. In addition, studies have shown that the mouthpiece adaptor used in topography measurement systems may interfere with nicotine transport, with the deposition of nicotine-containing aerosols on the inside of the additional tubing [41,42].

Compared to the self-reporting survey, video assessment, and puff-sensing devices, e-cigarettes with the a built-in puff recording feature are better positioned for naturalistic puff topography assessment, because (1) users can intuitively use the product with no interference or intervention from investigations and monitoring tools; (2) information about e-cigarette use, such as e-liquid and device power, can be simultaneously obtained in situ and integrated with puff topography to approximate user’s nicotine consumption; (3) users can view their own puffing data and track their own nicotine consumption in real time by connecting puff recording e-cigarettes with personal mobile devices, such as cell phones, via securely paired Bluetooth. This is especially valuable for those who want to gain the awareness to help quit or cut back nicotine use. By combining device-recorded puff data with laboratory nicotine emission results, the inhaled nicotine amount can theoretically be estimated, which otherwise must be done by collecting and analyzing multiple blood samples in a pharmacokinetic assessment. The application of e-cigarettes with a built-in puff recording feature provides a convenient and pragmatic avenue to bypass the nontrivial operations of blood specimen collection and thus makes it viable to directly assess the real-world nicotine use of e-cigarettes at large user population levels. In this report, a novel ENDS device with a built-in mechanism to record puff data (puff recording ENDS [PR-ENDS] device; VITRO (registered trademark), Shenzhen JWEI Electronics Co, Ltd) was investigated. We aimed to explore its feasibility in measuring puff topography during ad libitum use among smokers and vapers. The device’s capability in estimating nicotine consumption was further assessed based on comparison of the results calculated by the PR-ENDS device and blood sample analysis in nicotine pharmacokinetic assessment.

## Methods

### Participants

This is an ancillary study based on a completed parent study. The parent study was a 1-way randomized controlled open-label puff topography and nicotine pharmacokinetic assessment in 24 healthy adult volunteers who either regularly smoke combustible cigarettes (smoker group, n=12) or regularly vape e-cigarettes (vaper group, n=12). The participants were recruited in the Los Angeles, California metropolitan area in fall 2021. The study protocol and informed consent form (ICF) were reviewed and approved by the Institutional Review Board (IRB) (Advarra IRB). Potential participants were examined for study eligibility during the initial screening step. The individuals were included if they (1) were healthy males or females within the ages of 21 to 65 years; (2) were either current smokers (≥10 per day) of factory-made combustible cigarettes (eCO >10 ppm at screening) for at least 3 continuous months and occasional users of e-cigarettes, but none in the 14 days before the screening visit, or current daily users of e-cigarette devices with an e-liquid nicotine concentration >0 mg/mL (eCO ≤10 ppm at screening) for at least 3 continuous months and occasional users of combustible cigarettes, but none in the 14 days before the screening visit; and (3) had urine cotinine >200 ng/mL at screening.

### Study Design and Procedures

Following the screening step, participants visited the study site 6 times, including 1 visit with their own usual brand combustible cigarettes or e-cigarettes and 5 visits with PR-ENDS devices prefilled with assigned freebase nicotine e-liquids in a randomized product use sequence. Participants were provided with a supply of their assigned PR-ENDS product to use at home before their next visit for familiarization. Each individual visit was separated by a minimum washout period of 24 hours. During visits, each participant had 2 use sessions. In the first controlled use session, participants started by taking 10 puffs, 30 seconds apart, followed by a period of 120 minutes to allow the nicotine plasma concentration to ramp down to baseline. In the second ad libitum use session, participants were allowed to take as many puffs as desired during a period of 60 minutes. Throughout both the controlled use session and ad libitum use session, blood samples were obtained for plasma nicotine analysis, and subjective effect assessment questionnaires were administered at various time points. A schematic flowchart to describe the parent study is shown in Multimedia Appendix 1. Considering that the objective of the current investigation was to assess the naturalistic puff topography and nicotine consumption of the participating smokers and vapers, the ad libitum use session was the primary focus in this work, and the data collected from the ad libitum use session was used for the following analyses. The puff topography parameters (including number of puffs, total puff duration, and average puff duration) were measured by a CReSS device attached to the PR-ENDS device as a benchmark. Additionally, the puff data were recorded by the PR-ENDS device and analyzed after the parent investigation. It was registered as an ancillary study (with granted exemption by the IRB) to compare and examine the puff topography results.

### Materials and Measures

**Figure 1:**
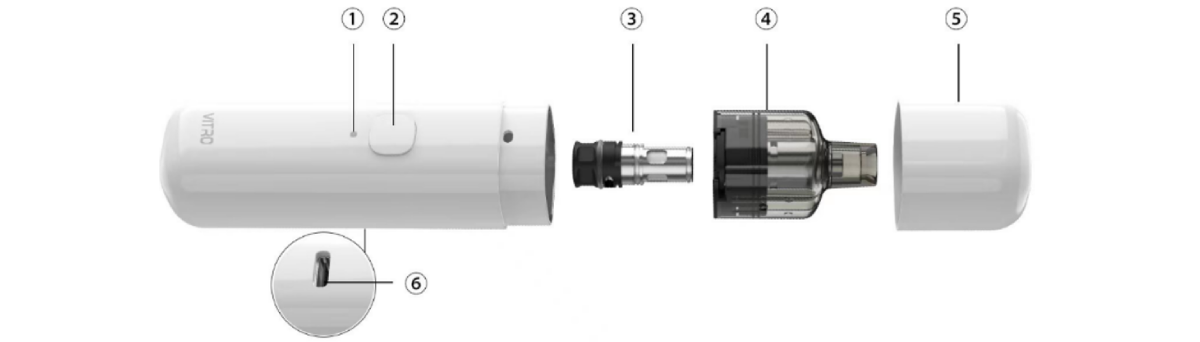
Exploded view of the key components of the puff recording electronic nicotine delivery system device. The components are as follows: (1) indicator light, (2) power button, (3) ProCore 0.8-ohm coil, (4) cartridge, (5) magnetic cover, and (6) charging port (type-C) on the back of the battery.

The investigated PR-ENDS device is an open refillable ENDS product with a removable 0.8-ohm coil and a 2-mL e-liquid capacity, and with 3 power settings (low power, 7-9 W; medium power, 9-11 W; and high power, 11-13 W). Figure 1 displays the exploded view of the PR-ENDS device.

One unique design feature of the PR-ENDS device is that it can measure puff data (including number of puffs, puff duration, and puff interval) through a built-in mechanism (button-activated puff recording) and via securely connected Bluetooth. The puff duration is measured and recorded based on the time of pressing and holding the power button. The specific scope of the investigated PR-ENDS device includes 2 power settings (high and low), 2 nicotine strengths (12 mg/mL and 3 mg/mL), and 2 flavors (tobacco and menthol). Detailed information about the investigated product groups (groups A-D) is shown in Table 1.

During the 1-hour ad libitum use session, e-liquid weight loss for assessment of nicotine consumption was calculated based on the weight difference before and after use sessions. Blood samples were taken from the study subjects at the following time points: 0 min, 30 min, and 60 min of 1-hour ad libitum use. Nicotine plasma concentrations at those time points were obtained based on nicotine analysis from blood samples. The area under the plasma concentration-time curve for 1-hour ad libitum use (AUC_ad lib_) was calculated based on the time course of the measured nicotine concentration from 0 min to 60 min.

**Table 1:** Description of the 5 investigated puff recording electronic nicotine delivery system device groups (A-E).

Product group	Flavor	Nicotine strength	Power
A	Tobacco	12 mg/mL	High
B	Menthol	12 mg/mL	High
C	Tobacco	12 mg/mL	Low
D	Tobacco	3 mg/mL	Low
E	Tobacco	3 mg/mL	High

### Statistical Analysis

Descriptive statistics, including means and SDs, were calculated for each variable. The box plots of targeted variables have been presented in figures. Two-sample *t* tests and paired *t* tests were applied to identify any statistically significant differences between compared samples. Variables were deemed significant at the level of 0.05 (α=.05). A 1-way or repeated measures ANOVA and Dunnett multiple comparison tests were conducted to assess stratified differences in variables among product use groups (groups A-E). The correlation coefficient (R^2^) was further calculated to assess the linear relationship between compared variables.

### Ethical Considerations

This ancillary study is an independent research topic that uses data from the completed parent study conducted in 2021. The parent study protocol and ICF were reviewed and approved by the IRB (Advarra IRB). This ancillary study was also reviewed by the IRB (Advarra IRB) and was granted exemption.

The IRB was constituted and operated in accordance with the principles and requirements described in the US Code of Federal Regulations (21 CFR Part 56). The IRB is compliant with the International Council for Harmonisation (ICH) guidelines.

The IRB granted exemption for informed consent in the secondary analysis in this ancillary study. In the parent study, all subjects voluntarily accepted to participate in this study and signed the ICF after having fully comprehended the contents of this form and before any study-specific procedures were performed. The identity of the subjects was kept confidential. Each eligible subject was assigned a subject number, which was used on the case report form instead of the subject’s name. Only the deidentified data were available for this ancillary study.

In the parent study, if the human subjects completed all 7 study visits, which included 6 testing visits, they were paid a total of US $1800 for participation at the completion of visit 7. The study participation payments were reported to the US Internal Revenue Service (IRS) as income. There was no payment for this ancillary study.

## Results

### Assessment of Puff Topography Measured by the PR-ENDS Device

The PR-ENDS device has the capability of recording the following 3 representative puff topography parameters: (1) number of puffs, (2) total puff duration (accumulated puffs within the 1-hour ad libitum use), and (3) average puff duration, for each individual user during product use. The puff durations measured by the PR-ENDS device have been proven consistent (with an accuracy within 0.1 second) by testing with preset puff duration parameters on puffing machines. Here, the presented assessment of puff topography provided further evidence for the puff recording feature in the PR-ENDS device.

As shown in Table 2, the number of puffs averaged 33.3 to 49.5 for groups A-E among smokers and 36.5 to 49.7 among vapers. Moreover, the total puff duration averaged 63.3 to 146.6 seconds for groups A-E among smokers and 77.1 to 133.4 seconds among vapers. Moreover, the average puff duration was 1.77 to 2.83 seconds for groups A-E among smokers and 1.97 to 2.60 seconds among vapers. Box plots of puff topography parameters measured by the PR-ENDS device are shown in Multimedia Appendix 2.

In the smoker group, the puff data recorded by the PR-ENDS device showed that higher device power (ie, group A vs group C and group E vs group D) and nicotine strength (ie, group A vs group E and group C vs group D) yielded lower values for the number of puffs, total puff duration, and average puff duration. Yet, a dissimilar trend was observed for the vaper group in that the power and nicotine strength only had limited effect on puff topography parameters. Such a difference might potentially indicate different puffing behaviors between smokers and vapers [35,37]. Interestingly, for both the smoker and vaper groups, the PR-ENDS device prefilled with menthol-flavored e-liquid yielded a lower number of puffs and lower total puff duration compared with tobacco-flavored e-liquid (group A vs group B). However, such a difference was not statistically significant (smokers: *P*=.25, vapers: *P*=.21) given the large variation in actual puff behavioral data.

**Table 2:** Descriptive summary of puff topography parameters measured by the puff recording electronic nicotine delivery system device.

Smoking category and product group		Number of puffs, mean (SD)	Total puff duration (seconds), mean (SD)	Average puff duration (seconds), mean (SD)
**Smoker**				
	Group A (tobacco/12/high^a^)	41.8 (20.2)	76.9 (43.7)	1.77 (0.59)
	Group B (menthol/12/high^a^)	33.3 (17.6)	63.3 (40.4)	1.84 (0.61)
	Group C (tobacco/12/low^a^)	44.5 (14.3)	101.3 (41.6)	2.23 (0.60)
	Group D (tobacco/3/low^a^)	49.5 (25.3)	146.6 (95.4)	2.83 (1.01)
	Group E (tobacco/3/high^a^)	45.4 (17.7)	113.2 (66.1)	2.39 (0.84)
**Vaper**				
	Group A (tobacco/12/high^a^)	42.3 (18.7)	101.0 (62.6)	2.15 (0.97)
	Group B (menthol/12/high^a^)	36.5 (13.7)	77.1 (46.8)	1.97 (0.85)
	Group C (tobacco/12/low^a^)	42.1 (14.4)	103.4 (53.8)	2.36 (0.62)
	Group D (tobacco/3/low^a^)	48.4 (24.0)	133.4 (87.9)	2.52 (0.84)
	Group E (tobacco/3/high^a^)	49.7 (21.9)	133.4 (72.3)	2.60 (1.22)

^a^The information in brackets indicates flavor/nicotine strength (mg/mL)/power.

### Comparison of Puff Topography Measurement Between the CReSS and PR-ENDS Devices

It is essential to validate the observed puff topography parameters measured by the PR-ENDS device. Although the CReSS device is not suited perfectly for analyzing e-cigarette puff topography [43], it is still considered to be one of the current standard approaches. Here, we employed a CReSS device to serve as the benchmark puff sensor to compare with the PR-ENDS device for recorded puff data. It should be noted that although the CReSS device is being widely used, it is known to have a low sensitivity for a low puff flow rate [36,41] and caution should be taken when assessing puff data from the CReSS device.

**Figure 2:**
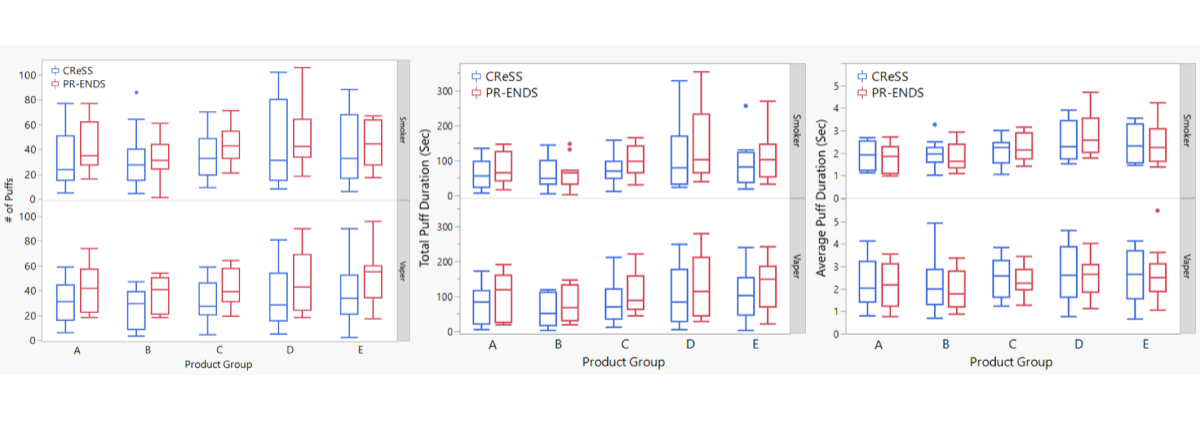
Comparison of the box plots of puff topography parameters measured by the Clinical Research Support System (CReSS) device (blue) and puff recording electronic nicotine delivery system (PR-ENDS) device (red). (A) Number of puffs, (B) total puff duration, and (C) average puff duration measured among smokers (top) and vapers (bottom). Group A, tobacco flavor/12 mg/mL nicotine strength/high power; Group B, menthol flavor/12 mg/mL nicotine strength/high power; Group C, tobacco flavor/12 mg/mL nicotine strength/low power; Group D, tobacco flavor/3 mg/mL nicotine strength/low power; Group E, tobacco flavor/3 mg/mL nicotine strength/high power.

As shown in Figure 2, the box plots of puff topography parameters demonstrated comparable values for the number of puffs, total puff duration, and average puff duration between the CReSS and PR-ENDS devices among smokers and vapers. Based on puff topography measured by the CReSS device (Multimedia Appendix 3), higher PR-ENDS device power and e-liquid nicotine strength were both associated with lower values of the number of puffs, total puff duration, and average puff duration in the smoker group, which are consistent with the observations made by the PR-ENDS device. In the vaper group, the correlation between device power/nicotine strength and puff parameters was less evident, which also concurred with the findings obtained by the PR-ENDS device as mentioned above.

In order to assess the comparability of puff topography parameters measured between the CReSS and PR-ENDS devices, statistical comparison tests were conducted. The results showed that no statistically significant differences were present based on the *P* values of 2-sample *t* test comparisons (Table 3), which indicated a strong agreement for the puff topography parameters measured between the PR-ENDS and CReSS devices at the population level. The findings also suggested that it would be feasible to use the PR-ENDS device as a noninterventional platform to assess users’ naturalistic puff topography, yielding the same level of puff recording sensitivity and accuracy as the CReSS device. Linear correlation analysis of puff topography parameters between the CReSS and PR-ENDS devices further showed that high correlation coefficients (R^2^) exist between data recorded by the CReSS and PR-ENDS devices (Multimedia Appendix 4, Multimedia Appendix 5, Multimedia Appendix 6).

**Table 3:** Two-sample *t* test comparisons of puff parameters measured between the Clinical Research Support System (CReSS) and puff recording electronic nicotine delivery system devices.

Smoking category and product group		*P* value for the number of puffs	*P* value for the total puff duration	*P* value for the average puff duration
**Smoker**				
	Group A (tobacco/12/high^a^)	.27	.36	.64
	Group B (menthol/12/high^a^)	.78	.95	.60
	Group C (tobacco/12/low^a^)	.41	.22	.52
	Group D (tobacco/3/low^a^)	.72	.32	.43
	Group E (tobacco/3/high^a^)	.57	.40	.99
**Vaper**				
	Group A (tobacco/12/high^a^)	.17	.48	.74
	Group B (menthol/12/high^a^)	.06	.41	.50
	Group C (tobacco/12/low^a^)	.12	.31	.95
	Group D (tobacco/3/low^a^)	.20	.42	.99
	Group E (tobacco/3/high^a^)	.28	.50	.97

^a^The information in brackets indicates flavor/nicotine strength (mg/mL)/power.

### PR-ENDS Puff Topography for Estimating Nicotine Consumption During Ad Libitum Use

Puff topography measurement provides a quantifiable base to estimate the amount of consumed nicotine. In theory, nicotine emission measured under certain puff topography (ie, method recommended by the Cooperation Centre for Scientific Research Relative to Tobacco [CORESTA]: 55-mL puff volume, 3-second puff duration, and 30-second puff interval [44]), combined with the number of puffs and puff duration in situ, can estimate how much nicotine gets aerosolized during a user’s inhalation process. On the other hand, nicotine consumption, defined as the amount of nicotine contained in e-liquid consumed by users [19], is an important factor in puff topography assessment. Nicotine consumption directly represents a user’s nicotine use, addiction, and abuse liability [45], especially when it is measured in an uncontrolled environment (ie, ad libitum use).

Previously, it has been recognized that nicotine emission in e-cigarette use is strongly impacted by applied device power [46] and e-liquid nicotine strength [38]. However, the e-cigarette nicotine use in an uncontrolled or natural environment (ie, ad libitum use) is largely unknown [21]. This is presumably due to the confounding effects from compensatory puffing behavior, as well as the large variation in puff topography within and across different individuals. For example, the “puff titration” effect has been observed in a previous study [47], where users tend to take more and longer puffs when freely using e-cigarettes with lower device powers and nicotine strengths. As such, “self-titration” of puffing behavior should increase the total puff duration so that it compensates the overall nicotine intake. With such a context, we intended to answer the following question: “Does lower device power and lower nicotine strength essentially lead to lower nicotine consumption, even considering the confounding effect from puff titration and compensatory behaviors?”

In order to prove the concept, nicotine consumption during the ad libitum use of a PR-ENDS device prefilled with designated e-liquids (groups A-E) was estimated. Specifically, nicotine consumption was calculated by integrating the PR-ENDS–measured puff topography data and laboratory nicotine emission results based on the following equation:

Nicotine consumption = (nicotine emission / testing puff duration) × total puff duration **(1)**

Specifically, nicotine consumption refers to the amount of nicotine consumed by PR-ENDS users during the ad libitum use session. The nicotine emission value (Multimedia Appendix 7) was obtained from laboratory testing with a puff regime of 3 seconds as the testing puff duration. In groups A-E, the nicotine emission had different values, as it was measured using a different device power (high or low), e-liquid nicotine strength (12 mg/mL or 3 mg/mL), and flavor (tobacco or menthol). The exact same power and e-liquid setup of the PR-ENDS device was used in laboratory testing to guarantee the reproducibility of the nicotine emission results. Total puff duration was obtained by either summing the individual puff duration of each puff recorded by the PR-ENDS device or directly reading from the PR-ENDS device built-in coil chip.

Nicotine consumption was calculated to represent how much nicotine has been consumed during the ad libitum use session. The assumption applied in the calculation is that nicotine consumption is projected as linearly proportional to the puff duration measured by the PR-ENDS device, which means it is not needed to involve puff volume or puff flow rate (puff volume over unit puff duration) into the calculation. This assumption is deemed reasonable, as a previous study [48] has clearly shown that the puff flow rate or puff volume does not impact aerosol emission yield and that puff duration alone is sufficiently representative for estimating the aerosolized nicotine generated from e-cigarettes.

**Figure 3:**
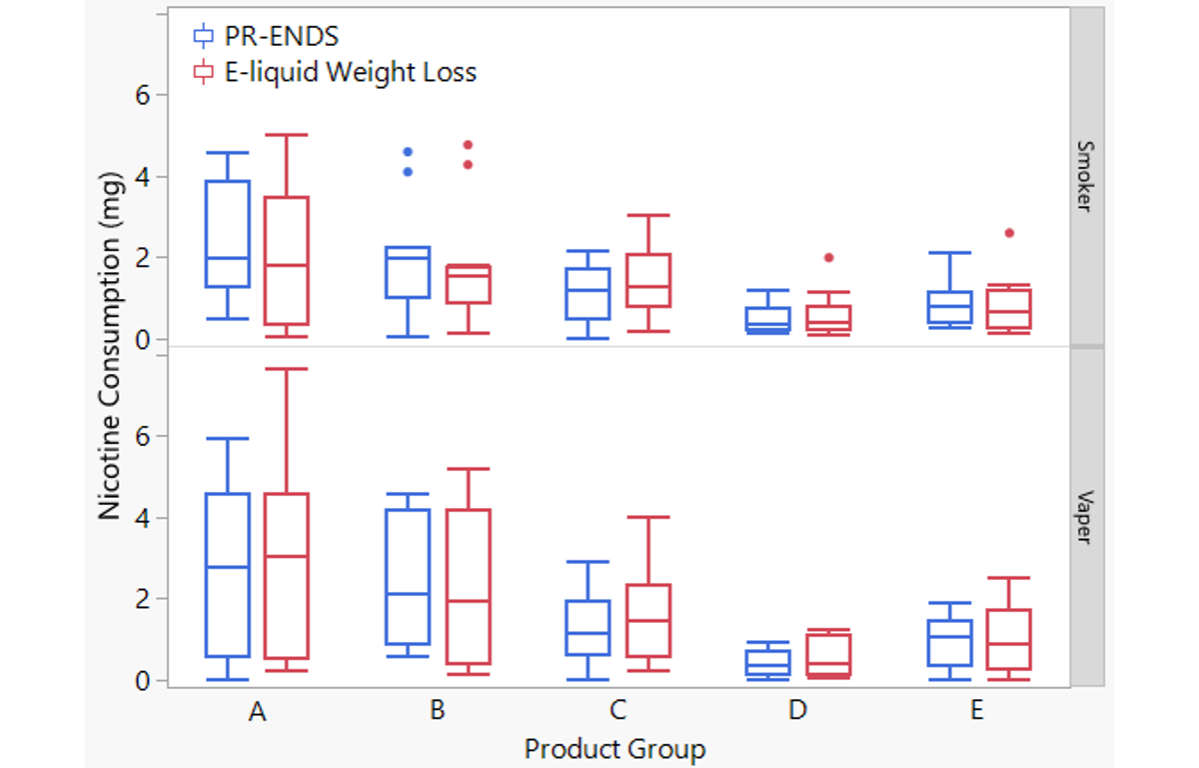
Comparison of the box plots of nicotine consumption within a 1-hour ad-libitum session between (1) value estimated based on puff topography measured by the puff recording electronic nicotine delivery system (PR-ENDS) device (blue) and (2) value estimated based on e-liquid weight loss (red) among smokers (top) and vapers (bottom). Group A, tobacco flavor/12 mg/mL nicotine strength/high power; Group B, menthol flavor/12 mg/mL nicotine strength/high power; Group C, tobacco flavor/12 mg/mL nicotine strength/low power; Group D, tobacco flavor/3 mg/mL nicotine strength/low power; Group E, tobacco flavor/3 mg/mL nicotine strength/high power.

In order to validate the assumption that nicotine consumption can be estimated based on the puff data recorded by the PR-ENDS device and laboratory aerosol emission results, the nicotine consumption value was alternatively obtained by calculating e-liquid weight loss multiplied by prelabeled nicotine concentrations. A similar calculation of nicotine consumption using e-liquid weight loss has been rationalized in a previous study [19]. As shown in Figure 3, the compared box plots of nicotine consumption between the PR-ENDS method and e-liquid weight loss method demonstrated that the 2 approaches yielded comparable values of nicotine consumption across groups A-E in smokers and vapers. The nicotine consumption derived from the PR-ENDS device showed average values of 0.48 to 2.40 mg for groups A-E among smokers and 0.40 to 2.63 mg among vapers. The nicotine consumption derived from e-liquid weight loss showed average values of 0.58 to 2.00 mg for groups A-E among smokers and 0.56 to 2.96 mg among vapers (Multimedia Appendix 8). In Table 4, two-sample *t* tests and paired *t* tests of nicotine consumption between the PR-ENDS and e-liquid weight loss methods showed that no statistically significant differences were present.

**Table 4:** Statistical comparison of nicotine consumption between the puff recording electronic nicotine delivery system device and e-liquid weight loss methods.

Smoking category and product group		Two-sample *t* test *P* value	Paired *t* test *P* value
**Smoker**			
	Group A (tobacco/12/high^a^)	.51	.45
	Group B (menthol/12/high^a^)	.68	.65
	Group C (tobacco/12/low^a^)	.34	.36
	Group D (tobacco/3/low^a^)	.56	.44
	Group E (tobacco/3/high^a^)	.82	.70
**Vaper**			
	Group A (tobacco/12/high^a^)	.70	.52
	Group B (menthol/12/high^a^)	.81	.67
	Group C (tobacco/12/low^a^)	.33	.06
	Group D (tobacco/3/low^a^)	.32	.07
	Group E (tobacco/3/high^a^)	.78	.54

^a^The information in brackets indicates flavor/nicotine strength (mg/mL)/power.

A repeated measures ANOVA was conducted to determine if there were statistical differences in nicotine consumption across the 5 PR-ENDS product groups (groups A-E). The results showed that for both smokers (*P*<.001) and vapers (*P*<.001), the nicotine consumption was significantly different among groups A-E. A further assessment with the Dunnett multiple comparisons test (control: group A) showed that device power (ie, group A vs group C) and nicotine strength (ie, group A vs group E) significantly impacted nicotine consumption (Table 5). More specifically, for both the smoker and vaper groups, higher device power (ie, group A vs group C and group E vs group D) and nicotine strength (ie, group A vs group E and group C vs group D) led to higher nicotine consumption during PR-ENDS device use, even though users could freely “puff titrate” the device (ie, puff more times or longer puffs) with lower device power and nicotine strength. As such, the PR-ENDS device demonstrated that by either limiting device power or nicotine strength, both can effectively reduce nicotine consumption. This conclusion has been reached based on the recorded puff data and laboratory nicotine emission results. Thus, there is no need to physically measure e-liquid weight loss to estimate nicotine use.

**Table 5:** Dunnett multiple comparisons test of nicotine consumption (estimated by puff topography parameters measured by the puff recording electronic nicotine delivery system device) among product groups A-E.

Smoking category and product group (comparison group: group A [tobacco/12/high^a^])		Nicotine consumption, mean difference (95% CI)	*P* value
**Smoker**			
	Group B (menthol/12/high^a^)	−0.4248 (−1.4207 to 0.5710)	.66
	Group C (tobacco/12/low^a^)	−1.2862 (−2.2820 to −0.2903)	.007
	Group D (tobacco/3/low^a^)	−1.5180 (−2.9125 to −0.9208)	<.001
	Group E (tobacco/3/high^a^)	−1.9167 (−2.5139 to −0.5222)	.001
**Vaper**			
	Group B (menthol/12/high^a^)	−0.2207 (−1.5569 to 1.1154)	.98
	Group C (tobacco/12/low^a^)	−1.3746 (−2.7107 to −0.0384)	.04
	Group D (tobacco/3/low^a^)	−1.6725 (−3.5588 to −0.8865)	<.001
	Group E (tobacco/3/high^a^)	−2.2226 (−3.0087 to −0.3364)	.009

^a^The information in brackets indicates flavor/nicotine strength (mg/mL)/power.

**Figure 4:**
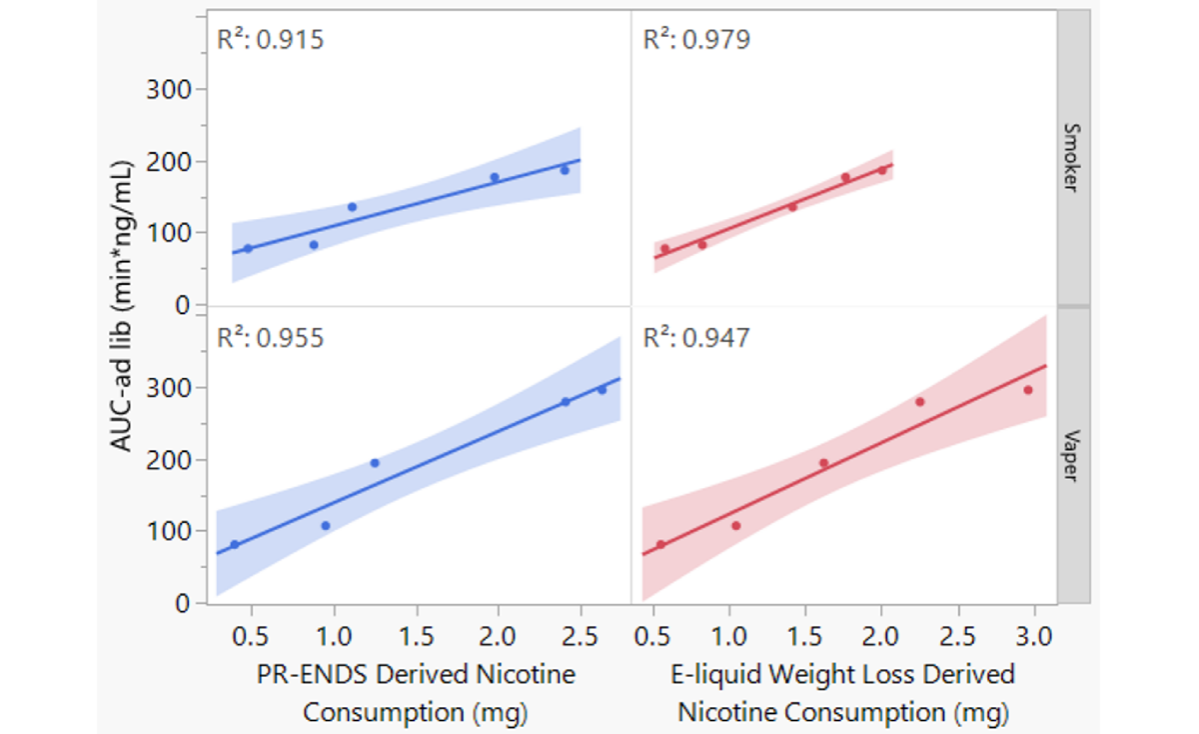
Linear regression of (1) AUC_ad lib_ and nicotine consumption (blue) derived from the puff recording electronic nicotine delivery system (PR-ENDS) device and (2) AUC_ad lib_ and nicotine consumption (red) derived from e-liquid weight loss among smokers (top) and vapers (bottom). The line represents the average, and the band represents the CI. AUC_ad lib_: area under the plasma concentration-time curve for 1-hour ad libitum use.

For clinical validation of nicotine consumption derived from the PR-ENDS device, the plasma nicotine concentration (blood samples collected at 0 min, 30 min, and 60 min) and AUC_ad lib_ during the 1-hour ad libitum use were calculated (Multimedia Appendix 9 and Multimedia Appendix 10). By definition, AUC represents the accumulated concentration of nicotine in blood samples over a certain period of time, which can be treated as a proxy of the intake of nicotine inhaled in the human body [49,50]. A linear regression analysis between the average nicotine consumption (from the PR-ENDS device) and the average pharmacokinetic parameter AUC_ad lib_ was conducted to determine if the nicotine consumption derived from the PR-ENDS device could be used to estimate nicotine intake. As shown in Figure 4, an almost perfect linear (R^2^=0.915-0.979) relationship between nicotine consumption derived from the PR-ENDS device and AUC_ad lib_ was present. Of note, a similar linear relationship was identified between nicotine consumption derived from e-liquid weight loss and AUC_ad lib_. These results, taken together, indicate that it is viable to use puff recording e-cigarettes, such as PR-ENDS devices, to directly assess puff topography, nicotine consumption, and intake in a natural use environment.

## Discussion

### Principal Findings

Despite the high relevance to nicotine abuse liability and inhalation health risk assessment, conducting noninvasive, accurate, and convenient measurements of e-cigarette puff topography is still challenging [32]. Various methods have been investigated to characterize e-cigarette puff topography; however, limitations still exist, such as unnaturalistic behaviors under investigations, large labor cost and time consumption (ie, video assessment), and unavoidable intervention for aerosol transportation in mouthpiece adaptors (ie, specialized puff-sensing devices such as CReSS). Conducting e-cigarette puff topography assessment in a naturalistic environment using current technologies is still considered difficult, and limited studies were reported for the naturalistic puffing behavior assessment [18,51].

The objective of this study was to evaluate the feasibility of a novel PR-ENDS device for measuring naturalistic puff topography and nicotine consumption during the ad libitum use of products. Based on the comparisons of puff numbers and puff durations measured between the PR-ENDS and CReSS (benchmark) devices, it has been clearly demonstrated that there is no significant populational difference in the recorded puff topography parameters between the 2 methods and across different device powers, e-liquid nicotine strengths, and flavors. The puff data recorded by the PR-ENDS device can be used to quantitatively estimate the nicotine consumption during natural use. This is achieved solely by integrating puff data with laboratory emission results in a mathematical model, with no need to physically collect e-liquid weight loss data. The linear relationship between nicotine consumption and the pharmacokinetic parameter AUC_ad lib_ provided further clinical validation of the noninvasive estimation of nicotine intake. Together, these results support the proposal that it is feasible to assess naturalistic puffing behavior and nicotine use with an e-cigarette having built-in puff recording features, such as a PR-ENDS device.

We found that the paired *t* tests of puff data from the CReSS and PR-ENDS devices indicated certain systematic discrepancies at the individual level (Multimedia Appendix 11). The CReSS device seemed to yield lower values compared to the PR-ENDS device for the number of puffs and total puff duration, but not for the average puff duration. This observation was particularly evident in the vaper group. Possible explanations for the observed puff difference between the CReSS and PR-ENDS devices are as follows: (1) the CReSS and PR-ENDS devices use different working mechanisms to record puffs (CReSS: flow-induced pressure drop; PR-ENDS: button-activation puff), which might introduce systematic variances in measurements (ie, users might push the button while not puffing and vice versa); (2) the CReSS device might be insensitive to low puff flow rates [36], which could lead to less records of the number of puffs and total puff duration; and (3) study participants might be unfamiliar with the PR-ENDS and CReSS devices, which could introduce inconsistent practice and human variations. These hypotheses need further investigations in future research.

Several observations were made during the puff topography assessment facilitated by the PR-ENDS device. First, device power and e-liquid nicotine strength were both observed to compensate users’ puffing behaviors, in that higher device power and nicotine strength led to fewer and shorter puffs during the ad libitum use, although such a discrepancy was less obvious among vapers than smokers. Based on puff data recorded by the PR-ENDS device, the average puff duration for e-liquids with a nicotine strength of 12 mg/mL (groups A, B, and C) was approximately 2.04 seconds, which is 21% shorter compared to 2.59 seconds for e-liquids with a nicotine strength of 3 mg/mL (groups D and E). Similar observations have been reported previously, and it has been shown that the use of lower nicotine strength e-liquids can increase the total puffed e-cigarette aerosol [38]. The average puff duration for high device power (groups A, B, and E) was approximately 2.11 seconds, which is 16% shorter compared to 2.50 seconds for low device power (groups C and D). As reported by a previous study [46], the number of puffs and puff duration were lower for third-generation e-cigarettes used at a higher power (10 W) than a lower power (6 W). In addition, menthol-flavored e-liquid used with the PR-ENDS device (group B) was found to be associated with fewer puffs and a shorter puff duration compared to tobacco-flavored e-liquid (group A), which supports the notion that flavored e-liquid can impact users’ puff topography. The e-liquid flavor effect on puffs in this study was observed to be different from the effect in a previous investigation, in which researchers found that the puff duration (analyzed by video) was the shortest for tobacco-flavored e-liquid and was the longest for menthol-flavored e-liquid among 5 different flavors [31].

Second, the PR-ENDS puff data indicated that the puffing behaviors of vapers are different from those of smokers. For example, vapers tended to have longer puffs than smokers based on the average puff duration (ie, in group A, smokers: 1.77 seconds; vapers: 2.15 seconds). This is consistent with published results [39], where researchers found that experienced e-cigarette users puff more intensively compared to naïve e-cigarette users (ie, smokers). The PR-ENDS device also identified that device power and nicotine strength tended to have less impact on the puff topography of vapers than smokers, which may presumably be explained by the finding that combustible cigarette smokers can be more susceptible to the switch from high nicotine products (ie, cigarettes) to low/medium nicotine products [35] (ie, PR-ENDS device prefilled with nicotine freebase e-liquids having low nicotine concentrations: 3/12 mg/mL), while vapers have already been using low/medium nicotine products so they are less susceptible to adjusting puffing behaviors. Further studies should be conducted to investigate these interpretations.

Third, nicotine consumption calculated from PR-ENDS puff data indicated that device power and nicotine strength could significantly influence nicotine consumption during ad libitum use, even when users could freely adjust and “self-titrate” their puff behaviors to compensate for the change in nicotine supplies. For example, the overall average nicotine consumption for e-liquids with nicotine strength of 12 mg/mL (groups A, B, and C) was approximately 1.96 mg, which is 188% higher compared to consumption of 0.68 mg for e-liquids with nicotine strength of 3 mg/mL (groups D and E). The overall average nicotine consumption for high device power (groups A, B, and E) was approximately 1.87 mg, which is 131% higher compared to consumption of 0.81 mg for low device power (groups C and D). In other words, higher nicotine strength and device power significantly boosted nicotine consumption within the scope of this study, even when we considered users’ puff compensatory behaviors. Furthermore, the relative impact on nicotine consumption from e-liquid nicotine strength was found to be bigger than device power. Such an observation is important, as it not only sheds light on the necessity of reducing the nicotine addictiveness of tobacco products, including the recently announced proposed rules by the Food and Drug Administration [52] of establishing a maximum level of nicotine in cigarettes, but also points out that e-liquids with a high nicotine strength unavoidably promote higher nicotine consumption. Regulations on e-liquid nicotine concentrations (ie, maximum nicotine concentration of 20 mg/mL in the European Tobacco Products Directive [TPD] [53]) should be further considered to reduce nicotine addiction risks.

### Limitations and Strengths

The current assessment was limited to 24 participants (12 smokers and 12 vapers). The small sample size caused a relatively large variation in puff measurements and thus hindered any further statistical analyses across different product groups. For example, no significant differences in puff topography parameters were recognized among groups A-E (Table 2). The 1-hour long ad libitum use of the PR-ENDS device was relatively short compared to the real-world use of e-cigarettes [51]. As a result, more long-term behavioral observations of the actual use of the PR-ENDS device should be conducted to understand users’ puffing behaviors in real-world conditions. Despite these limitations, the feasibility of using the PR-ENDS device for measuring naturalistic puff topography and nicotine consumption has been successfully demonstrated in this study.

There are a number of strengths. This is the first study to compare recorded puff topography parameters between an e-cigarette with a built-in puff recording feature and the CReSS device, a benchmark specialized puff-sensing device. Our results make an important contribution to the field of e-cigarette behavior research and provide a new avenue for assessing e-cigarette naturalistic puff topography and further derived analyses, such as nicotine consumption. With puff data readily accessible via a potentially paired smartphone app, we expect that the feasibility of the established PR-ENDS device in this report could inspire more research on the long-term real-time assessments of puff topography and nicotine consumption in real-world conditions, as well as more studies on the impact of empowered product use awareness (ie, smartphone app) on users’ puffing behaviors.

### Conclusion

In this study, we demonstrated the feasibility of a novel PR-ENDS device for assessing naturalistic puff topography and nicotine consumption during ad libitum use among smokers and vapers. The results proved the viability of using a PR-ENDS device for estimating the nicotine intake in the human body without drawing blood samples. Further, the potential effects of device power, nicotine strength, and e-liquid flavor, as well as differences between smokers and vapers on puff topography/behaviors and nicotine consumption were discussed. Together, the results presented in this study pave the way for future research with a focus on measuring naturalistic puff topography and behaviors in real-time and in real-world settings.

## Appendix

Multimedia Appendix 1 Schematic flowchart of the parent study.

Multimedia Appendix 2 Box plots of puff topography parameters measured by the puff recording electronic nicotine delivery system device.

Multimedia Appendix 3 Descriptive summary of puff topography parameters measured by the Clinical Research Support System device.

Multimedia Appendix 4 Linear correlations of measured number of puffs between the Clinical Research Support System and puff recording electronic nicotine delivery system devices.

Multimedia Appendix 5 Linear correlations of measured total puff duration between the Clinical Research Support System and puff recording electronic nicotine delivery system devices.

Multimedia Appendix 6 Linear correlations of measured average puff duration between the Clinical Research Support System and puff recording electronic nicotine delivery system devices.

Multimedia Appendix 7 Summary of the average nicotine emission per puff for the puff recording electronic nicotine delivery system device.

Multimedia Appendix 8 Descriptive summary of nicotine consumption estimated by puff topography parameters measured by the puff recording electronic nicotine delivery system device and e-liquid weight loss.

Multimedia Appendix 9 Descriptive summary of nicotine pharmacokinetic parameters.

Multimedia Appendix 10 Nicotine plasma concentration-time profile over 60 minutes by product group among smokers and vapers.

Multimedia Appendix 11 Paired t-test comparison of puff topography parameters measured between the Clinical Research Support System and puff recording electronic nicotine delivery system devices.

## Abbreviations

AUC: area under the curve
AUC_ad lib_: area under the plasma concentration-time curve for 1-hour ad libitum use
CReSS: Clinical Research Support System
ENDS: electronic nicotine delivery system
ICF: informed consent form
IRB: Institutional Review Board
PR-ENDS: puff recording electronic nicotine delivery system

## Data Availability

The data sets generated during and/or analyzed during this study are not publicly available as the puff recording electronic nicotine delivery system proprietary data and summary are currently undergoing the Food and Drug Administration evaluation process, but are available from the corresponding author on reasonable request.
