# Supplementary material for: A Novel Puff Recording Electronic Nicotine Delivery System for Assessing Naturalistic Puff Topography and Nicotine Consumption During Ad Libitum Use: Ancillary Study
Source: JMIR Form Res. 2023 Jan 16;7:e42544. doi: 10.2196/42544 (PMC9887514; doi:10.2196/42544)

**Multimedia Appendix 2.** Box plots of puff topography parameters measured by the puff recording electronic nicotine delivery system device. (a) Number of puffs, (b) total puff duration, (c) average puff duration.


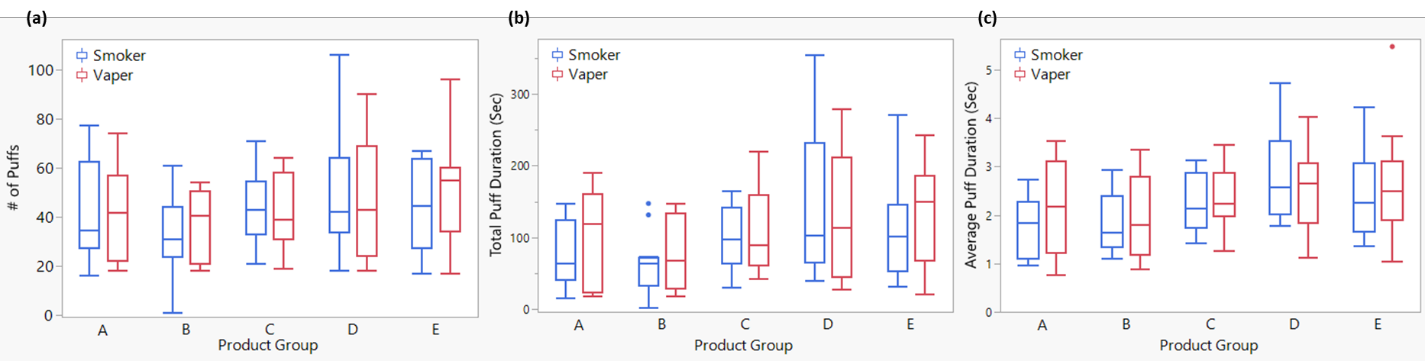

Supplement: Multimedia Appendix 2 [file formative_v7i1e42544_app2.docx]
