# Supplementary material for: A Novel Puff Recording Electronic Nicotine Delivery System for Assessing Naturalistic Puff Topography and Nicotine Consumption During Ad Libitum Use: Ancillary Study
Source: JMIR Form Res. 2023 Jan 16;7:e42544. doi: 10.2196/42544 (PMC9887514; doi:10.2196/42544)
Supplement: Multimedia Appendix 3 [file formative_v7i1e42544_app3.docx]

**Multimedia Appendix 3.** Descriptive summary of puff topography parameters measured by the Clinical Research Support System device.

|  | **Product Group** | **# of puffs** | **Total puff duration (second)** | **Average puff duration (second)** |
| --- | --- | --- | --- | --- |
| Smoker | A (Tobacco/12/High) | 31.8 (22.2) | 60.3 (41.6) | 1.88 (0.60) |
|  | B (Menthol/12/High) | 30.9 (23.5) | 62.1 (45.2) | 1.97 (0.58) |
|  | C (Tobacco/12/Low) | 38.1 (18.9) | 77.8 (39.5) | 2.05 (0.57) |
|  | D (Tobacco/3/Low) | 44.9 (34.8) | 106.9 (91.1) | 2.52 (0.88) |
|  | E (Tobacco/3/High) | 40.0 (26.2) | 89.5 (68.2) | 2.39 (0.79) |
| Vaper | A (Tobacco/12/High) | 30.5 (18.2) | 80.5 (60.2) | 2.31 (1.19) |
|  | B (Menthol/12/High) | 25.0 (15.0) | 61.0 (44.9) | 2.27 (1.22) |
|  | C (Tobacco/12/Low) | 31.4 (16.9) | 77.7 (60.0) | 2.38 (0.85) |
|  | D (Tobacco/3/Low) | 34.8 (24.4) | 101.8 (88.2) | 2.52 (1.17) |
|  | E (Tobacco/3/High) | 38.9 (21.4) | 110.7 (72.8) | 2.58 (1.04) |

Note: Puff topography parameters measured by the PR-ENDS device (# of puffs, total puff duration, and Average puff duration) are demonstrated in mean (SD).
