# Supplementary material for: A Novel Puff Recording Electronic Nicotine Delivery System for Assessing Naturalistic Puff Topography and Nicotine Consumption During Ad Libitum Use: Ancillary Study
Source: JMIR Form Res. 2023 Jan 16;7:e42544. doi: 10.2196/42544 (PMC9887514; doi:10.2196/42544)

**Multimedia Appendix 5.** Linear correlations of measured total puff duration between the Clinical Research Support System and puff recording electronic nicotine delivery system devices.


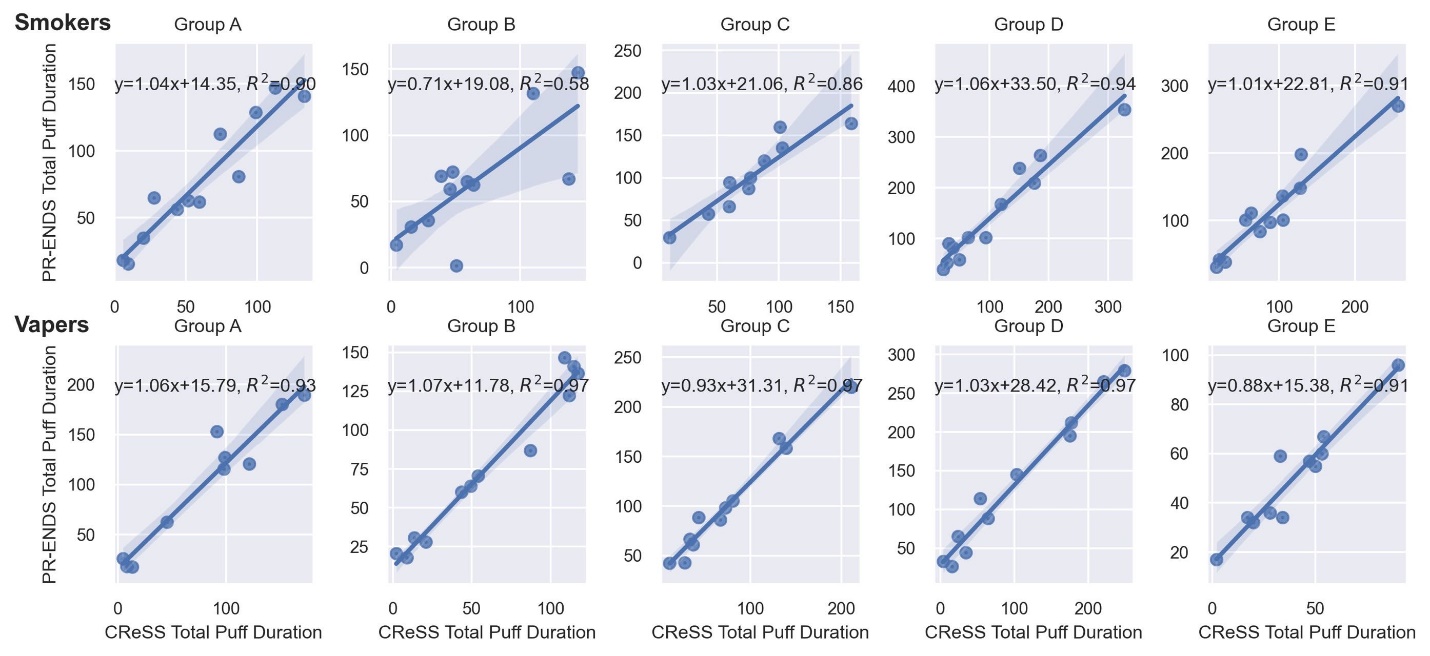

Supplement: Multimedia Appendix 5 [file formative_v7i1e42544_app5.docx]
