# Supplementary material for: A Novel Puff Recording Electronic Nicotine Delivery System for Assessing Naturalistic Puff Topography and Nicotine Consumption During Ad Libitum Use: Ancillary Study
Source: JMIR Form Res. 2023 Jan 16;7:e42544. doi: 10.2196/42544 (PMC9887514; doi:10.2196/42544)
Supplement: Multimedia Appendix 7 [file formative_v7i1e42544_app7.docx]

**Multimedia Appendix 7.** Summary of the average nicotine emission per puff for the puff recording electronic nicotine delivery system device.

| **Device power** | **Puff duration*** | **Nicotine concentration of e-liquid (mg/ml)** | **Average nicotine emission (mg/puff)**** |
| --- | --- | --- | --- |
| High | 3 second | 3 | 0.0180 |
|  | 3 second | 12 | 0.0952 |
| Medium | 3 second | 3 | 0.0144 |
|  | 3 second | 12 | 0.0762 |
| Low | 3 second | 3 | 0.0111 |
|  | 3 second | 12 | 0.0406 |

*The puff duration from laboratory testing was set as Coresta recommendation method (CRM) with 55ml volume, 3 second puff duration, and 30 second puff intervals.

**The average is calculated from 21 replicates (n = 21).
