# Supplementary material for: A Novel Puff Recording Electronic Nicotine Delivery System for Assessing Naturalistic Puff Topography and Nicotine Consumption During Ad Libitum Use: Ancillary Study
Source: JMIR Form Res. 2023 Jan 16;7:e42544. doi: 10.2196/42544 (PMC9887514; doi:10.2196/42544)
Supplement: Multimedia Appendix 8 [file formative_v7i1e42544_app8.docx]

**Multimedia Appendix 8.** Descriptive summary of nicotine consumption estimated by puff topography parameters measured by the puff recording electronic nicotine delivery system device and e-liquid weight loss.

|  | **Product Group** | **PR-ENDS Derived Nicotine Consumption (mg)** | **E-liquid Weight Loss Derived Nicotine Consumption (mg)** |
| --- | --- | --- | --- |
| Smoker | A (Tobacco/12/High) | 2.40 (1.42) | 2.00 (1.72) |
|  | B (Menthol/12/High) | 1.98 (1.32) | 1.76 (1.40) |
|  | C (Tobacco/12/Low) | 1.11 (0.74) | 1.42 (0.91) |
|  | D (Tobacco/3/Low) | 0.48 (0.33) | 0.58 (0.55) |
|  | E (Tobacco/3/High) | 0.88 (0.54) | 0.83 (0.68) |
| Vaper | A (Tobacco/12/High) | 2.63 (2.23) | 2.96 (2.36) |
|  | B (Menthol/12/High) | 2.41 (1.53) | 2.25 (1.80) |
|  | C (Tobacco/12/Low) | 1.25 (0.81) | 1.62 (1.16) |
|  | D (Tobacco/3/Low) | 0.40 (0.32) | 0.56 (0.47) |
|  | E (Tobacco/3/High) | 0.95 (0.64) | 1.05 (0.84) |

Note: The estimated nicotine consumptions are demonstrated in mean (SD).
