# Supplementary material for: A Novel Puff Recording Electronic Nicotine Delivery System for Assessing Naturalistic Puff Topography and Nicotine Consumption During Ad Libitum Use: Ancillary Study
Source: JMIR Form Res. 2023 Jan 16;7:e42544. doi: 10.2196/42544 (PMC9887514; doi:10.2196/42544)
Supplement: Multimedia Appendix 9 [file formative_v7i1e42544_app9.docx]

**Multimedia Appendix 9.** Descriptive summary of nicotine pharmacokinetic parameters.

|  | **Product Group** | **C_0min_ (ng/mL)** | **C_30min_ (ng/mL)** | **C_60min_ (ng/mL)** | **AUC_ad lib_ (min*ng/mL)** |
| --- | --- | --- | --- | --- | --- |
| **Smoker** | A (Tobacco/12/High) | 3.59 (2.11) | 6.41 (3.50) | 8.35 (5.60) | 186 (121) |
|  | B (Menthol/12/High) | 3.44 (2.32) | 6.31 (3.99) | 7.46 (4.98) | 177 (155) |
|  | C (Tobacco/12/Low) | 2.74 (1.76) | 4.90 (1.89) | 5.89 (2.45) | 135 (112) |
|  | D (Tobacco/3/Low) | 2.02 (1.32) | 3.02 (1.62) | 3.39 (1.97) | 77.8 (76.1) |
|  | E (Tobacco/3/High) | 1.85 (1.44) | 3.78 (2.69) | 3.98 (2.49) | 82.6 (58.1) |
| **Vaper** | A (Tobacco/12/High) | 5.04 (6.47) | 7.53 (7.45) | 11.34 (9.83) | 295 (302) |
|  | B (Menthol/12/High) | 3.90 (4.38) | 7.04 (5.98) | 9.35 (7.88) | 279 (307) |
|  | C (Tobacco/12/Low) | 4.12 (6.15) | 6.80 (6.72) | 8.34 (6.91) | 194 (129) |
|  | D (Tobacco/3/Low) | 2.58 (3.11) | 3.67 (3.34) | 3.95 (2.98) | 81.1 (75.4) |
|  | E (Tobacco/3/High) | 3.36 (4.70) | 4.99 (5.94) | 6.12 (6.73) | 107 (94.9) |
