# Supplementary material for: A Novel Puff Recording Electronic Nicotine Delivery System for Assessing Naturalistic Puff Topography and Nicotine Consumption During Ad Libitum Use: Ancillary Study
Source: JMIR Form Res. 2023 Jan 16;7:e42544. doi: 10.2196/42544 (PMC9887514; doi:10.2196/42544)

**Multimedia Appendix 10.** Nicotine plasma concentration-time profile over 60 minutes by product group among smokers (top) and vapers (bottom). The line represents the average, and the error bar represents the standard error.


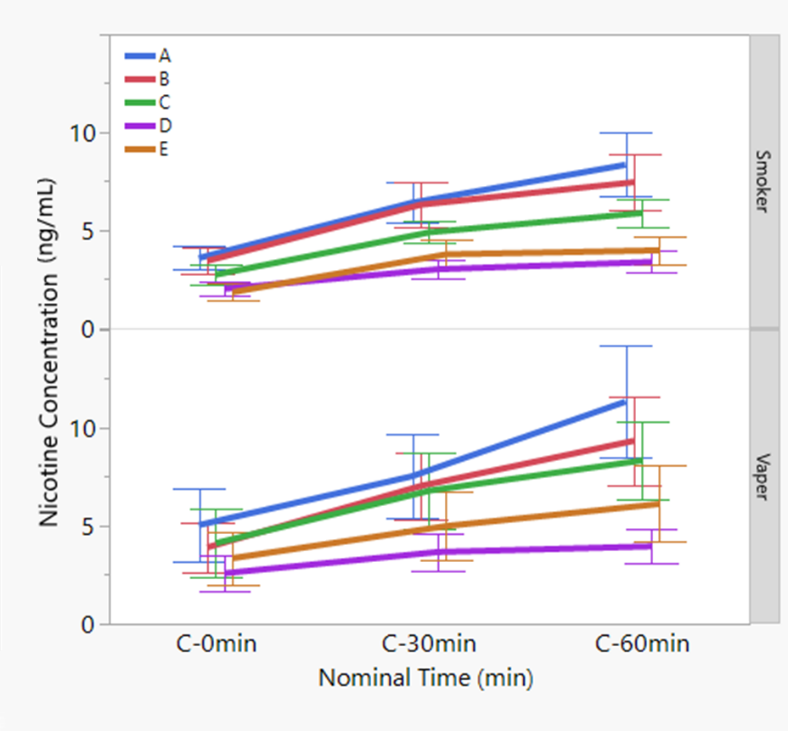

Supplement: Multimedia Appendix 10 [file formative_v7i1e42544_app10.docx]
