# Supplementary material for: A Novel Puff Recording Electronic Nicotine Delivery System for Assessing Naturalistic Puff Topography and Nicotine Consumption During Ad Libitum Use: Ancillary Study
Source: JMIR Form Res. 2023 Jan 16;7:e42544. doi: 10.2196/42544 (PMC9887514; doi:10.2196/42544)
Supplement: Multimedia Appendix 11 [file formative_v7i1e42544_app11.docx]

**Multimedia Appendix 11.** Paired *t*-test comparison of puff topography parameters measured between the Clinical Research Support System and puff recording electronic nicotine delivery system devices.

| **Comparison between CReSS and PR-ENDS device** | | **Paired t-test, *P* value** | | |
| --- | --- | --- | --- | --- |
|  | **Product Group** | **# of puffs** | **Total puff duration** | **Average puff duration** |
| **Smoker** | A (Tobacco/12/High) | <.001 | .002 | .40 |
|  | B (Menthol/12/High) | .56 | .89 | .36 |
|  | C (Tobacco/12/Low) | .07 | .001 | .36 |
|  | D (Tobacco/3/Low) | .32 | <0.001 | .28 |
|  | E (Tobacco/3/High) | .15 | .002 | .98 |
| **Vaper** | A (Tobacco/12/High) | <0.001 | .004 | .24 |
|  | B (Menthol/12/High) | <0.001 | <0.001 | .09 |
|  | C (Tobacco/12/Low) | <0.001 | <0.001 | .87 |
|  | D (Tobacco/3/Low) | <0.001 | <0.001 | .99 |
|  | E (Tobacco/3/High) | <0.001 | <0.001 | .92 |
